# Evaluating continuum of maternal and newborn healthcare in Rwanda: evidence from the 2019–2020 Rwanda demographic health survey

**DOI:** 10.1186/s12884-022-05109-9

**Published:** 2022-10-19

**Authors:** Quraish Sserwanja, Ghislaine Gatasi, Milton W. Musaba

**Affiliations:** 1 Programmes Department, GOAL Global, Khartoum, Sudan; 2 Key Laboratory of Environmental Medicine Engineering, School of Public Health, Ministry of Education, Southeast University, 210009 Nanjing, Jiangsu Province China; 3 Department of Obstetrics and Gynaecology, Mbale Regional Referral and Teaching Hospital, Mbale, Uganda; 4 Department of Obstetrics and Gynaecology, Busitema University, Tororo, Uganda

**Keywords:** Continuum of care, Antenatal care, Postnatal care, Skilled birth attendance, Women and Rwanda

## Abstract

**Background:**

Access to a complete continuum of maternal and child health care has been recommended globally for better pregnancy outcomes. Hence this study determined the level (pooled prevalence) and predictors of successfully completing continuum of care (CoC) in Rwanda.

**Methods:**

We analyzed weighted secondary data from the 2019–2020 Rwanda Demographic and Health Survey (RDHS) that included 6,302 women aged 15 to 49 years who were selected using multistage stratified sampling. We analyzed complete continuum of care as a composite variable of three maternal care services: at least four ANC contacts, SBA, maternal and neonatal post-natal care. We used the SPSS version 25 complex samples package to conduct multivariable logistic regression.

**Results:**

Of the 6,302 women, 2,131 (33.8%) (95% CI: 32.8–35.1) had complete continuum of care. The odds of having complete continuum of care were higher among women who had exposure to newspapers (adjusted odds ratio (AOR): 1.30, 95% CI: 1.11–1.52), those belonging to the eastern region (AOR): 1.24, 95% CI: 1.01–1.52), southern region (AOR): 1.26, 95% CI: 1.04–1.53), those with health insurance (AOR): 1.55, 95% CI: 1.30–1.85), those who had been visited by a field health worker (AOR: 1.31, 95% CI: 1.15–1.49), those with no big problems with distance to health facility (AOR): 1.25, 95% CI: 1.07–1.46), those who were married (AOR): 1.35, 95% CI: 1.11–1.64), those with tertiary level of education (AOR): 1.61, 95% CI: 1.05–2.49), those belonging to richer households (AOR): 1.33, 95% CI: 1.07–1.65) and those whose parity was less than 2 (AOR): 1.52, 95% CI: 1.18–1.95).

**Conclusion:**

We have identified modifiable factors (exposure to mass media, having been visited by a field health worker, having health insurance, having no big problems with distance to the nearest health facility, belonging to richer households, being married and educated), that can be targeted to improve utilization of the entire continuum of care. Promoting maternity services through mass media, strengthening the community health programmes, increasing access to health insurance and promoting girl child education to tertiary level may improve the level of utilization of maternity services.

**Supplementary Information:**

The online version contains supplementary material available at 10.1186/s12884-022-05109-9.

## Background

Globally, almost all neonatal deaths (75%) occur in the first week of life [1]. Majority of the global maternal and neonatal deaths occur in low and middle income countries (LMICs) with sub-Saharan Africa (SSA) having the largest burden and the least access to the complete continuum of maternal care [2, 3]. Furthermore, 98% of the global stillbirth burden occurs in LMICs with 75% occurring in SSA and south Asia [4].Timely access to antenatal care (ANC), skilled birth attendance (SBA) and postnatal (PNC) care are key evidence based strategies that have been proven to ensure a reduction in stillbirths, maternal and neonatal mortality and morbidity [5–7]. Accordingly, the world health organization (WHO) highly advocates for the implementation of continuum of maternal and newborn care which ensures continual access to care from preconception through to the postpartum period [8, 9].

Sustainable Development Goal (SDG) three targets to reduce maternal and neonatal mortality to less than 70 deaths per 100 000 livebirths and 12 per 1000 livebirths respectively [10]. As much as they sound ambitious, they reaffirmed the global prioritization of maternal child health (MCH). However, achieving this will need a high universal coverage of the components of maternal and newborn care for early detection of complications to enable timely intervention [10–12]. Therefore, a clear understanding of the utilization behavior of the components of maternal and newborn continuum of care is vital not only towards reducing inequity in health but also towards designing and implementing better strategies aimed at improving the unacceptably poor MCH indicators [13].

Although the 1994 genocide devastated Rwanda’s health system, efforts were put in place to ensure that the system is rebuilt which partly contributed to the country’s achievement of the Millennium-um Development Goals (MDG) 4 of reducing the under-five mortality rate by two-thirds and MDG 5 of reducing the maternal mortality ratio by three-quarters [14–16]. However, Rwanda has not had similarly sized reductions in neonatal mortality compared to non-neonatal under-5 mortality [17, 18] with neonatal mortality rate (NMR) currently at 19 deaths per 1,000 live births and contributing over 42% of under 5 mortality [19]. Furthermore, although Rwanda reduced its the maternal mortality ratio (MMR) from 750 deaths per 100,000 live births to 210 deaths per 100,000 live births within a decade (2005–2015), the MMR has stagnated in the last five years from 210 to 203 deaths per 100,000 live births [19, 20].

The 2020 Rwanda Demographic and Health Survey (RDHS) reports variation in maternal care utilization with only 47% of the women accessing at least four ANC contacts compared to SBA of 94% [19]. Given that WHO modified the minimum number of ANC visits from four to at least eight contacts in 2016, the current ANC utilization in Rwanda is far behind the global recommendations [21–23]. Therefore, in order for Rwanda to reduce the MMR to less than 70 per 100,000 live births and the NMR to less than 12 per 1000 live births by 2030, up-to-date data is needed to further guide the ongoing maternal and newborn health projects. Studies that have looked at continuum of care (CoC) have combined Rwanda’s DHS data with other countries ending up with results that cannot be specific to Rwanda [13, 24]. Hategeka et al. only looked at single measures (ANC and place of delivery), PNC, SBA were not considered in this study [25]. Therefore, we used the most recent nationally representative survey data to determine level and predictors of successfully completing CoC.

## Methods

### Setting

Rwanda is a central-eastern African nation of about 12 million people [19, 25] whose health system consists of eight national referral hospitals, four hospitals at provincial level, 35 district level hospitals, 495 health centers, 406 health posts and over 45,000 community health workers (CHWs) [20, 26, 27]. Each village has a male-female CHW pair and one female *Agent de Sante Maternelle* (ASM) and these CHWs are responsible in delivering the first line of health services including maternal and newborn health services [20, 28]. The country has a universal, community-based health insurance program that has a household subscription and co-payments at the time of care and all citizens are eligible to enroll into it [20, 29].

### Study sampling and participants

The 2019-20 Rwanda Demographic Survey (RDHS) was used for this analysis employed a two-stage sample design with the first stage involving sample points (clusters) selection consisting of enumeration areas (EAs) [19]. Between November 2019 and July 2020, a total of 13,005 households were selected from the first stage selected EAs [19]. The household and the woman’s questionnaires provided the data used in this secondary analysis.

Eligibility for women to participate in the RDHS was being aged between 15 and 49 years and being either permanent residents of the selected households or visitors who stayed in the household the night before the survey [19]. Out of the total 13,005 households originally sampled, 12,951 had occupants and 12,949 were interviewed [19]. We included only women who had given birth within five years preceding the survey in this analysis. Out of the 14,675 women found eligible for the RDHS, the team was able to interview 14,634 women of which only 6,302 women had given birth within the last five years preceding the survey [19].

### Variables

#### Outcome variables

Complete continuum of maternal and newborn healthcare was coded 1 and incomplete coded as 0 [6]. Complete continuum was considered as having utilized all the three maternal healthcare services; at least four ANC contacts, SBA, at least one maternal and neonatal PNC checkup within six weeks after childbirth [6, 30, 31].

#### Independent variables

Nineteen independent variables were categorized into women and household characteristics, and were chosen basing on previous studies [32–34] and availability in the RDHS database as shown in Table 1.

**Table 1:** Categorization of independent variables

Variable	Categorization
Exposure to newspapers or magazines	Yes, and No
Exposure to internet	Yes and No
Exposure to radio	Yes and No
Exposure to television (TV)	Yes and No
Access to internet	Yes and No
Age	15–24 years, 25–34 years and 35–49 years
Residence	Urban and Rural
Region	North, East, South, West and Kigali
Household size	Less than 6 and 6 and above. (this was based on the average household size of 5)
Sex of household head	Male and Female
Parity	1, 2–4, 5, and above
Level of education	No education, primary, secondary, and tertiary (highest level of education attended)
Working status	Yes and No
Wealth index	Richest, richer, middle, poorer, and poorest quintiles
Having health insurance	Yes and No
Having been visited by a field health worker within the last 12 months	Yes and No
Problems seeking permission to get medical care	No big problems and big problems (RDHS had three original categories (no problem, no big problem and big problem) however, after data collection, no woman reported no problem)
Problems with distance to nearest health facility	No big problems and big problems (RDHS had three original categories (no problem, no big problem and big problem) however, after data collection, no woman reported no problem)
Marital status	Married/cohabiting and Not married
Year of birth	2014–2015, 2016, 2017, 2018, 2019 and 2020 (we combined 2014 with 2015 because 2014 only had 9 births)

### Statistical analysis

We used the complex sample package of SPSS (version 25.0) statistical software which accounted for the multi-stage cluster study design [35]. Individual sample weight, sample strata In order to ensure representativeness of the survey results at the national and regional level and to minimize the effects of unequal probability sampling in different strata, data were weighted [12]. Initially, we did descriptive statistics. Frequencies and proportions/percentages for categorical variables have been presented. We then used bivariable logistic regression to assess the association between each independent variable and the outcome whose crude odds ratio (COR), 95% confidence interval (CI) and p-values have been presented. Independent variables found significant at p-value less than 0.25 at bivariable level with other independent variables were considered for multivariable logistic regression to assess the independent effect of each variable on the CoC utilisation [6]. Before multivariable analysis, multicollinearity was assessed using variance inflation factor (VIF) and no VIF above 2.5 was observed. Model fitness was assessed with the F-test with a p-value of < 0.001. Adjusted odds ratios (aOR), 95% confidence intervals (CI) and p-values were calculated with statistical significance level set at p-value < 0.05.

## Results

A total of 6,302 women were included in the analysis (Table 2). Of these, 2,131 (33.8%) (95% CI: 32.8–35.1) had complete continuum of care (further details in Table 3 and Supplementary file 1). Only 16 (0.3%) of the women were able to utilize at least eight ANC contacts. Majority of the women had less than four ANC contacts (52.8%), had had skilled birth attendance (94.3%), had a postnatal check (70.7%), Furthermore, majority of the women were aged 20 to 34 years (63.0%), had primary education (64.5%), had no exposure to internet (89.3%), were married (80.8%), working (75.7%) and resided in rural areas (82.2%). The mean age and household size were 31.56 ± 6.74 years and 5.14 ± 1.86 members respectively.

**Table 2:** Socio-demographic characteristics of women who gave birth within the last 5 years prior to the 2020 RDHS

Characteristics	N = 6,302	%
**Age**		
35 to 49	2207	35.0
20 to 34	3970	63.0
15 to 19	125	2.0
**Household size**		
6 and above	2357	37.4
Less than 6	3945	62.6
**Exposure to newspapers/magazines**		
No	5070	80.5
Yes	1232	19.5
**Exposure to radio**		
No	1448	23.0
Yes	4854	77.0
**Exposure to TV**		
No	3753	59.5
Yes	2549	40.5
**Sex of Household head**		
Male	4783	75.9
Female	1519	24.1
**Internet access**		
No	5626	89.3
Yes	676	10.7
**Wealth Index**		
Poorest	1448	23.0
Poorer	1217	19.3
Middle	1224	19.4
Richer	1234	19.6
Richest	1178	18.7
**Parity**		
1	1587	25.2
2–4	3550	56.3
5 and above	1165	18.5
**Marital**		
Not married	1208	19.2
Married/cohabiting	5094	80.8
**Visited by a fieldworker**		
No	3994	63.4
Yes	2307	36.6
**Has health insurance**		
No	1194	18.9
Yes	5108	81.1
**Working status**		
Not working	1532	24.3
Working	4770	75.7
**Permission to access healthcare**		
Big problem	222	3.5
Not big problem	6080	96.5
**Distance to health facility**		
Big problem	1461	23.2
Not big problem	4841	76.8
**Region**		
North	1004	15.9
East	1702	27.0
West	1425	22.6
South	1305	20.7
Kigali	866	13.7
**Residence**		
Rural	5179	82.2
Urban	1123	17.8
**Education Level**		
No Education	698	11.1
Primary Education	4071	64.5
Secondary Education	1258	20.0
Tertiary Education	275	4.4
**Year of birth**		
2014	9	0.1
2015	556	8.8
2016	932	14.8
2017	1292	20.5
2018	1588	25.3
2019	1633	25.9
2020	292	4.6

**Table 3:** Utilization of the different components of continuum of care

Service	Frequency N = 6,302	%	95% CI
**4 or more ANC contacts**	2975	47.2	46.1–48.6
**Skilled birth attendance**	5950	94.3	93.9–95.0
**Maternal PNC**	4456	70.7	69.7–72.0
**Neonatal PNC**	4815	76.4	75.5–77.6
**Continuum of care**	2131	33.8	32.8–35.1

### Factors associated with CoC utilisation

Factors associated with CoC utilisation are shown in Table 4. Women who had exposure to newspapers (adjusted odds ratio (AOR): 1.30, 95% CI: 1.11–1.52) those belonging to the eastern region (AOR): 1.24, 95% CI: 1.01–1.52), southern region (AOR): 1.26, 95% CI: 1.04–1.53), those with health insurance (AOR): 1.55, 95% CI: 1.30–1.85), those who had been visited by a field health worker (AOR: 1.31, 95% CI: 1.15–1.49), those with no big problems with distance to health facility (AOR): 1.25, 95% CI: 1.07–1.46), those who were married (AOR): 1.35, 95% CI: 1.11–1.64), those with tertiary level of education (AOR): 1.61, 95% CI: 1.05–2.49), those belonging to richer households (AOR): 1.33, 95% CI: 1.07–1.65) and those whose parity is less than 2 (AOR): 1.52, 95% CI: 1.18–1.95) were more likely to utilize complete continuum of care. Women belonging to the western region (AOR): 0.76, 95% CI: 0.61–0.94) were less likely to utilize complete continuum of care.

**Table 4:** Factors associated with CoC utilization

Characteristics	crude model cOR (95% CI)	P-value	Adjusted model aOR (95% CI)	P-value
**Age**				
35 to 49	1		1	
25 to 34	1.12 (0.98–1.27)	0.097	0.92 (0.79–1.08)	0.301
15 to 24	0.89 (0.59–1.36)	0.600	0.75 (0.48–1.17)	0.203
**Household size**				
6 and above	1		1	
Less than 6	1.27 (1.12–1.43)	< 0.001	1.12 (0.96–1.29)	0.150
**Exposure to newspapers/magazines**				
No	1		1	
Yes	1.84 (1.59–2.13)	< 0.001	1.30 (1.11–1.52)	0.001
**Exposure to radio**				
No	1		1	
Yes	1.62 (1.39–1.89)	< 0.001	1.15 (0.96–1.38)	0.137
**Exposure to TV**				
No	1		1	
Yes	1.50 (1.32–1.71)	< 0.001	1.15 (0.99–1.34)	0.075
**Sex of Household head**				
Male	1		1	
Female	0.84 (0.73–0.96)	0.011	0.99 (0.85–1.17)	0.967
**Internet**				
No	1		1	
Yes	2.06 (1.70–2.49)	< 0.001	1.30 (0.98–1.73)	0.66
**Wealth Index**				
Poorest	1		1	
Poorer	1.28 (1.06–1.55)	0.010	1.18 (0.97–1.44)	0.101
Middle	1.52 (1.26–1.83)	< 0.001	1.34 (1.09–1.64)	0.005
Richer	1.67 (1.39–2.02)	< 0.001	1.33 (1.07–1.65)	0.009
Richest	2.01 (1.64–2.47)	< 0.001	1.21 (0.93–1.57)	0.161
**Parity**				
5 and above	1		1	
2–4	1.57 (1.32–1.86)	< 0.001	1.41 (1.15–1.74)	0.001
Less than 2	1.66 (1.36–2.03)	< 0.001	1.52 (1.18–1.95)	0.001
**Married/cohabiting**				
No	1		1	
Yes	1.42 (1.22–1.65)	< 0.001	1.35 (1.11–1.64)	0.002
**Visited by a fieldworker**				
No	1		1	
Yes	1.35 (1.19–1.52)	< 0.001	1.31 (1.15–1.49)	< 0.001
**Health insurance**				
No	1		1	
Yes	1.99 (1.69–2.34)	< 0.001	1.55 (1.30–1.85)	< 0.001
**Working**				
**No**	1		-	
**Yes**	0.93 (0.81–1.07)	0.308		
**Permission to access healthcare**				
Big problem	1		1	
Not big problem	1.80 (1.29–2.52)	0.001	1.33 (0.92–1.91)	0.129
**Distance to health facility**				
Big problem	1		1	
Not big problem	1.43 (1.24–1.66)	< 0.001	1.25 (1.07–1.46)	0.004
**Region**				
North	1		1	
East	1.22 (0.99–1.48)	0.052	1.24 (1.01–1.52)	0.037
West	0.73 (0.59–0.91)	0.006	0.76 (0.61–0.94)	0.013
South	1.25 (1.03–1.52)	0.023	1.26 (1.04–1.53)	0.021
Kigali	1.09 (0.84–1.42)	0.500	0.85 (0.66–1.11)	0.240
**Residence**				
Rural	1		-	
Urban	1.09 (0.92–1.29)	0.330		
**Education level**				
None	1		1	
Primary	1.27 (1.03–1.56)	0.024	0.95 (0.77–1.18)	0.663
Secondary	1.58 (1.23–2.02)	< 0.001	0.92 (0.69–1.21)	0.528
Tertiary	3.82 (2.75–5.30)	< 0.001	1.61 (1.05–2.49)	0.031
**Year of Birth**				
2014–2015	1		1	
2016	1.12 (0.90–1.40)	0.310	1.11 (0.88–1.41)	0.378
2017	0.95 (0.77–1.16)	0.593	0.95 (0.76–1.18)	0.624
2018	1.15 (0.93–1.42)	0.211	1.16 (0.92–145)	0.208
2019	1.19 (0.95–1.49)	0.125	1.11 (0.88–1.41)	0.383
2020	1.16 (0.83–1.61)	0.381	0.95 (0.67–1.33)	0.759

## Discussion

The current study reveals that only a third of Rwandan women received all elements of the continuum of care, and these women tended to be those from the Eastern region, those exposed to mass media (newspapers), those who had been visited by a field health worker, those who are from richer households, having tertiary level of education, married, low parity those with insurance and those with no big problems with distance to the nearest health facility.

The prevalence of complete continuum of care in this study is lower than findings from Cambodia, Egypt and Zambia [6, 31, 36] and higher than findings from studies conducted in Pakistan, Tanzania, Ethiopia, Ghana and Uganda [30, 37–40]. This low CoC finding is surprising because Rwanda is one of the countries in SSA with comprehensive national health insurance [41]. However, it is evident from this study that ANC utilization is low given that less than half (47.2%) of women are able to utilize at least four ANC contacts and this contributed to less completion of CoC.

Exposure to mass media (newspapers/magazines) showed a significant association with CoC utilization with the exposed women having higher odds of CoC. This finding is not surprising because several similar studies from other countries within in the region have reported this positive association between utilisation of mass media and the utilisation of MCH services [6, 7, 42, 43]. Gugsa et al. while examining newspaper coverage of maternal health in Bangladesh, Rwanda and South Africa showed that unlike South Africa, Rwanda was on track having the second highest maternal health coverage having increased from 15 articles to 158 in four years with 69% of the articles having a ‘human-rights’ or ‘policy-based’ frame [44].The influence of media on ensuring increased utilization of maternal health services including ANC, SBA and PNC could be linked to the role health information broadcasted on mass media play in ensuring reduction of maternal health knowledge shortage by sensitizing women and their partners on the benefits of timely access to care leading to positive attitudes and improves health seeking behavior [6, 45, 46]. In addition, women who are exposed to newspapers and television are more likely to be educated/literate and are in better position to have heath literacy related discussions with others which may further contribute to the challenging negative norms that might affect health seeking and hence lead to positive health seeking behavioral change [6, 47, 48].

Women who had been visited by a field health worker had higher odds of CoC utilization. Rwanda’s health system has a strong community health based programme [49]. Each village in Rwanda consists of three community health workers (CHWs) including one female Agent de Sante Maternelle (ASM) and a male–female pair (Binômes) [28]. Maternal care is provided by the ASMs through home and follow-up visits and referrals. CHWs are supported by the health ministry through the nearest health centres and are provided with a kit that has the needed supplies and a prepaid mobile phone to ease communication with the CHW cell coordinators [28]. In addition to the trainings supported by the government, some non-governmental organizations provides additional training and financial support to the CHWs [28].

These field based health workers are crucial in ensuring access to maternal care especially in areas with shortage of health facilities and large distances needed to be covered by women. The field health workers help equip mothers with knowledge on the dangers of using unskilled birth attendants and complications of pregnancies in addition to providing care, referring the complicated cases and also encouraging them to seek care within health facilities where possible [50]. Furthermore, these field health workers are being used to help track the progress of women along the continuum of care and follow-up defaulters [24]. Being visited by field health workers has been shown to be associated with maternal healthcare service utilization in several other studies [24, 51–53].

Women in the eastern region had higher odds of CoC utilization. This could be partly attributed to the high focus of national and international non-governmental organizations (NGOs) in the eastern region after it experienced some of the worst effects of the genocide that negatively affected the health status [14, 15]. These NGOs focused mainly on maternal and newborn care, worked closely with the ministry of health (MOH) and provided highly effective support that included; direct care provision, ensuring adequate and qualified staff, infrastructure development, robust quality improvement mechanisms and financial and technical assistance to support clinical innovation areas [14, 15]. Likewise, the smoother landscape found in eastern provinces compared to the northern region could be attributed to a higher likelihood of MCH service uptake because it enables for easier access to health facilities, as other studies have shown [54, 55]. The odds of having CoC in the western region were less. Despite the government’s initiatives to strengthen its healthcare system, the western region has lagged behind, with the majority of the population still lacking access to primary health care and more than half of the facilities operating under capacity [56]. Huerta et al. while assessing ***“geographical accessibility and spatial coverage modeling of the primary health care network in the Western Province”*** showed that when considering walking as the single mode of transportation, less than a third of the western province population is covered by the catchment area of the existing primary health facility network [56]. Fadelu et al. when assessing access to tertiary cancer care further showed that geographically, the most underserved region was the western one as none (0%) of the population were able to access care within an hour [57]. Hence there is high need for maternal stakeholders to target access to care in the western region.

Women who had no big problems with distance to the nearest health facility had higher odds of CoC utilization. Distance to health facilities has been found elsewhere in similar contexts to be an important determinant of maternal healthcare services utilization [11]. Given that most women ( 82.2% in this study) reside in rural areas which tend to have shortage of health facilities hence have to cover longer distances with poor road infrastructure [58]. This leads them to incur indirect transport costs or cover long distances and those that are unable to afford these transport costs or cover the long distances will end up attending less or no ANC contacts, have unskilled birth attendance and have no PNC [54, 58, 59]. Distance doesn’t only affect women but also community health workers who provide care in the community [54]. Thus, Rwanda’s maternal healthcare stakeholders need to explore sustainable solutions to health facility access barriers such as maternity waiting homes and construction of more health facilities.

Women with health insurance had higher odds of CoC utilization a finding that has been shown in other studies [60–63]. Rwanda’s community-based health insurance (CBHI) program is locally coordinated at the district level with enrollment being promoted by the village mobilization committees at the village level [61, 64, 65]. The program is funded by donor and development partners, the government, and individual contributions with prenatal and postnatal care being covered in the minimum package [29, 61]. Pregnant women incur several hidden expenses which may arise from the long distances covered when accessing care, buying medicines and supplies when they are out of stock in public facilities leading to high out-of-pocket (OOP) expenditures which tend to have a negative effect on access to maternal health services [63, 66]. Health insurance coverage reduces the rates of out-of-pocket payments associated with the use of MHC services utilization hence can improve health seeking and utilization behaviors [60, 63].

Women belonging to richer households had more odds of having CoC compared to those from the poorest households a finding consistent with several SSA studies [43, 67, 68]. Women belonging to households with limited financial resources may be unable to afford the medical and non-medical costs involved in seeking maternal care [43, 69, 70]. This might lead to those women not seeking maternal care, seeking late or limiting the number of ANC contacts which negatively affects completion of CoC [43]. Although Rwanda has a very good national health insurance scheme, not all women have subscribed to it as evidenced by over 18% of women in this analysis having no insurance hence some women still pay out of pocket for direct maternal healthcare costs and even those that have insurance, many still have to pay for the indirect costs such as transportation. This implies that lower levels of wealth can also be a barrier to accessing maternal health services hence multi-sectoral actors and different attentions are needed to eliminate financial barriers to improve the continuity of maternity care in Rwanda.

Consistent with findings from Ethiopia, Ghana and Uganda, increased levels of education have been shown to be associated with higher odds of completing CoC [40, 71, 72]. Educated women are more likely to have better maternal health literacy, more likely to have better paying jobs hence increased ability to afford direct and indirect costs associated with accessing maternal care [73]. Furthermore, highly educated women have been shown to be more receptive to new maternal health related information, increased knowledge of availability and location of maternal health resources and increased decision-making abilities which factors are crucial to ensure positive health seeking behaviour [71, 74].

Married women had higher odds of having CoC compared to their non-married counterparts. Marital status’s positive association with utilization and outcomes of maternal healthcare has been documented in several countries [43, 75–78]. Marriage provides better planning/desirability of care, societal acceptability, more psychosocial and financial support to women from their partners and in laws which support provides women with more time to access care and ability to afford the direct and indirect costs involved in accessing maternal care [43, 75, 79, 80]. Furthermore, the psychosocial support from partners, partner’s friends and in laws means understanding, sympathy and communication, which can positively motivate the women to seek timely and adequate maternal care [80].

Women with less parity had higher odds of having CoC compared to those whose parity was five and above. Increased utilization of maternal healthcare services among women with less parity has been shown in several other SSA countries [81–84]. This could be partly attributed to the fact that women with more children believe they are experienced with pregnancy and childbirth hence they are less anxious about the possibility of negative outcomes hence utilize maternal healthcare services late and less compared to those with lower parity [43, 81–83].

## Conclusion

Only 3 out of 10 women were able to utilize the entire continuum of maternity care services. Regarding ANC utilization, less than half (47.2%) and only 0.3% of women were able to utilize at least four and eight ANC contacts respectively a finding showing that Rwanda is not on track towards achieving the recommended eight ANC contacts for each pregnant woman in the 2016 WHO ANC guidelines. Belonging to eastern and southern Rwanda, exposure to mass media, having been visited by a field health worker, having health insurance, having no big problems with distance to the nearest health facility, belonging to richer households, having tertiary level of education, being married and having low parity were positively associated with completion of maternal and newborn continuum of care. Thus, we recommend the different stakeholders engaged in maternal health care to focus on women from western region, those without insurance, from poor households, with low levels of education, high parity and unmarried to ensure utilization of complete continuum of maternal and newborn care. Promoting maternity services through mass media, strengthening the community health programmes, increasing access to health insurance, strengthening and promoting girl child education to post-secondary level, increased support to household economic development programmes may improve the level of utilization of maternity services. Utilization of mass media specially to sensitize the population on the benefits of continuum of care should be encouraged with great attention given for early initiation of ANC, increasing the number of ANC contacts, health facility and skilled birth attendance during childbirth and postnatal care. During ANC contacts in health facilities and in the community by CHWs unmarried and high parity women need to be given more time during care, awareness on the importance of having CoC and followed up actively.

## Electronic supplementary material

Below is the link to the electronic supplementary material.

**Supplementary Material 1**

## Data Availability

The data that support the findings of this study are available from MEASURE DHS website (URL: https://www.dhsprogram.com/data/available-datasets.cfm) but restrictions apply to the availability of these data, which were used under license for the current study, and so are not publicly available. Data are however available from the MEASURE DHS website (URL: https://www.dhsprogram.com/data/available-datasets.cfm) upon reasonable request and with permission of MEASURE DHS.

## Abbreviations

EA: Enumeration area.
AOR: Adjusted Odds Ratio.
CI: Confidence Interval.
COR: Crude Odds Ratio.
DHS: Demographic Health Survey.
RDHS: Rwanda Demographic Health Survey.
OR: Odds Ratio.
SD: Standard Deviation.
WHO: World Health Organization.
ANC: Antenatal care.
PNC: Postnatal care.
SBA: Skilled Birth Attendance.
CoC: Continuum of Care.
SPSS: Statistical Package for Social Science.
