# Supplementary material for: Evaluating continuum of maternal and newborn healthcare in Rwanda: evidence from the 2019–2020 Rwanda demographic health survey
Source: BMC Pregnancy Childbirth. 2022 Oct 19;22:781. doi: 10.1186/s12884-022-05109-9 (PMC9583497; doi:10.1186/s12884-022-05109-9)
Supplement: Supplementary file 1 — Supplementary Material 1 [file 12884_2022_5109_MOESM1_ESM.docx]

**Frequency of continuum of care among women in Rwanda as per the 2020 RDHS**

| Continuum of care | Frequency | Percentage |
| --- | --- | --- |
| 0 service | 239 | 3.8 |
| 1 service | 624 | 9.9 |
| 2 services | 877 | 13.9 |
| 3 services | 2432 | 38.6 |
| 4 services | 2131 | 33.8 |
